# Finding patterns in cortical responses

**DOI:** 10.7554/eLife.56234

**Published:** 2020-04-23

**Authors:** Alessandro Sanzeni, Mark H Histed

**Affiliations:** 1 Department of Neurobiology, Duke UniversityDurhamUnited States; 2 Intramural Research Program, National Institute of Mental HealthBethesdaUnited States

**Keywords:** specific connectivity, inhibitory-stabilised networks, patterned perturbation, Mouse

## Abstract

Simulations predict a paradoxical effect that should be revealed by patterned stimulation of the cortex.

**Related research article** Sadeh S, Clopath C. 2020. Patterned perturbation of inhibition can reveal the dynamical structure of neural processing. *eLife* **9**:e52757. doi: 10.7554/eLife.52757

Any system, including biological systems, can be said to perform a computation when it transforms input information to generate an output. It is thought that many brain computations are performed by neurons (or groups of neurons) receiving input signals that they process to produce output activity, which then becomes input for other neurons. Many computations that brains can perform could, in principle, be carried out through feedforward processes (Yamins et al., 2014). In simple terms, feedforward means that the signals always travel in one direction – forward to the next neuron or network of neurons – and they never travel backwards or sideways to other neurons within a neuron group. In the cortex, however, networks of neurons have substantial 'recurrent' connectivity. Most cortical neurons are connected to other nearby cortical neurons, and therefore, signals can travel sideways due to these recurrent, local connections.

One property of networks with recurrent connectivity is that they can amplify certain inputs to produce larger outputs, while suppressing other inputs or amplifying them by a smaller factor. However, it has been challenging to understand how this can happen without the system displaying unstable or runaway activity, which is undesirable in the brain because it can lead to epileptic seizures. One plausible mechanism for recurrent amplification is known as 'balanced amplification' (Murphy and Miller, 2009). In mathematical network models that support balanced amplification, recurrent connectivity allows certain inputs to produce large outputs, yet the networks still exhibit other properties that are consistent with experimental data (such as fast responses to inputs). Recurrent connections can also influence the timing of neurons’ responses, allowing shorter inputs to create long-lasting, or time-varying outputs (Hennequin et al., 2014).

Neurons can be excitatory or inhibitory: when an excitatory neuron fires, the neuron receiving that input becomes more likely to fire as well, and when an inhibitory neuron fires, the opposite occurs, and the recipient neuron is suppressed. A network of excitatory and inhibitory cells must possess strong recurrent connectivity to support many recurrent computations, including balanced amplification. Here 'strong' means that recurrent connections are sufficiently dense to allow excitatory neurons to amplify other excitatory neurons’ activity, and in this situation, strong inputs from inhibitory neurons are required to stop the network from becoming unstable. More precisely, inhibitory-stabilized network models are those where, if the activity of inhibitory neurons could be locked to a fixed level, the excitatory neurons in the network would then become unstable (Tsodyks et al., 1997). Inhibitory-stabilized networks have been found in several cortical areas, and are seen across a range of levels of network activity – both when sensory stimulation is present, and when it is absent (Ozeki et al., 2009; Li et al., 2019; Sanzeni et al., 2019, but see Mahrach et al., 2020).

The simplest form of strong connectivity amongst excitatory neurons in a network is where the whole excitatory network is unstable. This is the standard inhibitory-stabilized network. But complex neural networks can have multiple unstable excitatory modes, where subgroups of excitatory neurons are unstable and would display runaway behavior if they were not stabilized by inhibition. Networks in which inhibition stabilizes multiple excitatory modes or subgroups are said to be in detailed balance (Vogels and Abbott, 2009; Hennequin et al., 2014; Litwin-Kumar and Doiron, 2014), while those in which inhibition stabilizes a single group of excitatory cells, typically the group of all excitatory cells, are in global balance. As a general rule, networks in detailed balance are also in global balance.

Now, in eLife, Sadra Sadeh and Claudia Clopath from Imperial College London report the result of simulations that show that networks in detailed balance have properties that extend the basic inhibitory-stabilized network (Sadeh and Clopath, 2020). In globally-balanced networks, when inhibitory neurons are stimulated uniformly (all of the neurons across the network receive an input of the same strength) a distinctive ‘paradoxical’ response, where adding input reduces activity, can be observed (Figure 1C). These paradoxical responses can be used as a signature to determine whether the network is an inhibitory-stabilized network (Tsodyks et al., 1997). Sadeh and Clopath extend this idea to detailed-balance networks with multiple unstable excitatory modes. They show that if the inhibitory neurons in these networks receive more complex, patterned stimulation (that is, certain neurons receive a stronger input than others) a predictable paradoxical signature can be observed (Figure 1D). Sadeh and Clopath call networks in which this happens ‘specific inhibitory-stabilized networks’. The connectivity patterns between neurons in their models are consistent with anatomical evidence of structured network connectivity in the cortex (Ko et al., 2013; Znamenskiy et al., 2018). Further, the existence of multiple excitatory submodes in the cortex is suggested by recent experiments that have found preferential amplification of specific patterns of input (Marshel et al., 2019; Peron et al., 2020).

**Figure 1.**
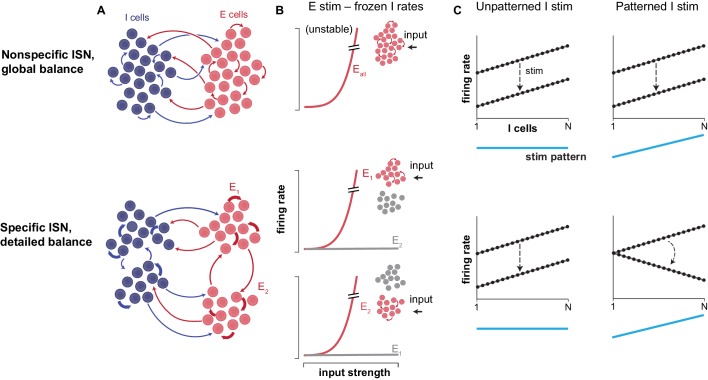
Inhibitory structure revealed by patterned stimulation. (**A**) Two possible network structures create two types of inhibitory-stabilized networks (ISNs). In a non-specific network (top), any excitatory (or inhibitory) neuron (E cell or I cell) has the same probability of connecting with other excitatory (inhibitory) neurons. In a specific network, subgroups of neurons connect preferentially to other neurons within the subgroup. (**B**) The two types of network require two different kinds of inhibitory balance. These types of balance are illustrated here conceptually, via a thought experiment where one imagines inhibitory neurons’ (I cells’) activity is frozen at a fixed level. For non-specific networks (top), if inhibitory neurons’ activity could be frozen, a single group of excitatory cells would respond to input (x-axis) by entering runaway behavior together. Thus, during normal network operation, feedback from inhibitory neurons is required to stabilize this single excitatory mode. The network is then said to be in global balance. For specific networks (bottom), multiple excitatory modes (subgroups of E neurons) are unstable when inhibition is frozen. During normal network operation, the inhibitory network must be connected in such a way as to stabilize these multiple excitatory modes, and these networks are said to be in detailed balance. (**C**) Sadeh and Clopath examine how firing rates (y-axis) of different inhibitory cells (x-axis) change when stimulated, depending on whether the stimulation pattern (blue line) was uniform (left panels) or patterned (right panels) in non-specific (top) or specific (bottom) networks (note that the specific networks that Sadeh and Clopath simulated have even more than two excitatory modes; see their work for details). The dotted lines show inhibitory firing rates, before (upper line) and after (lower line) stimulation, with the change in firing rates induced by stimulation indicated by the arrow. In both specific and non-specific networks, stimulation that excites the inhibitory neurons uniformly (left) paradoxically leads to a decrease in their firing rates. Patterned stimulation of inhibitory cells (right) in non-specific networks (top) leads to a similar response as with uniform inputs. However, in specific networks (bottom), patterned stimulation adds another effect: the inhibitory neurons that receive the strongest stimulation decrease their activity the most.

Sadeh and Clopath thus make a concrete prediction: that this “specific paradoxical effect” will be seen in networks where the connectivity between neurons is strong and structured. This prediction can now be tested using a technique called two-photon optogenetics that allows patterned input to be provided to neural networks in vivo with single-cell resolution, both for excitatory and inhibitory neurons (for example, Marshel et al., 2019; Forli et al., 2018).

The article by Sadeh and Clopath also takes a conceptual step forward by considering the information that can be gained about network structure and function by providing each neuron with an input of different strength. This conceptual framework is timely, as two-photon stimulation has this ability to vary the strength of the input to selected neurons. Specifically, Sadeh and Clopath predict that a pattern of input across inhibitory neurons will generate a response that is similar to the input pattern but with opposite sign. These predictions should shape future experiments, yielding new information about a key element of cortical function: how the recurrent connectivity in cortical networks is used for computation.
